# Factors associated with clinical outcomes of pediatric dengue shock syndrome admitted to pediatric intensive care unit: A retrospective cohort study

**DOI:** 10.1016/j.amsu.2021.102472

**Published:** 2021-06-06

**Authors:** Syifa Armenda, Desy Rusmawatiningtyas, Firdian Makrufardi, Eggi Arguni

**Affiliations:** Department of Child Health, Faculty of Medicine, Public Health and Nursing, Universitas Gadjah Mada/Dr. Sardjito Hospital, Yogyakarta, 55281, Indonesia

**Keywords:** Predictor factor, Clinical outcome, Pediatric ICU, Dengue shock syndrome

## Abstract

**Background:**

Dengue shock syndrome (DSS) in children is a challenging infectious disease due to its high mortality rate. Several factors can contribute to the DSS patients’ outcomes. Here we defined factors associated with clinical outcomes of patients with DSS in Pediatric Intensive Care Unit (PICU).

**Methods:**

We retrospectively collected data from January 2016 to May 2020 of patients who had been diagnosed with DSS and admitted to PICU in our tertiary referral hospital. Bivariate analysis and logistic regression were used to evaluate independent predictors of the study outcomes.

**Results:**

Overall, 146 patients were enrolled in this study, including 53.4% males and 46.6% females. The mortality rate during the study period was 5.5%. Fluid overload percentage, shock condition at PICU admission, DIC, and AKI were independent predictors for DSS mortality in logistic regression test with p < 0.05. There were several factors correlated with prolonged stay, including disseminated intravascular coagulation (DIC) (RR 15.26; 95% CI: 4.97–46.81), and nutritional status (RR 16.47; 95% CI: 3.72–72.9).

**Conclusion:**

Fluid overload percentage, shock condition at PICU admission, DIC, and AKI are independent predictors for DSS mortality. Several factors contribute to prolonged PICU stay, including DIC and nutritional status.

## Highlights

• Dengue shock syndrome is still a major problem in childrens' health in Indonesia.
• Infection and fluid overload should to be concerned to prevent prolonged PICU stay.
• The importance of increasing referral response times from previous hospitals in dengue shock syndrome cases.

## 1 Introduction

Dengue infection is an endemic disease in Indonesia that causes a significant impact on the public health and economy [1]. A study in a pediatric intensive care unit (PICU) showed that 23% of patients with dengue shock syndrome (DSS) became deceased and 49.2% had prolonged hospitalization. Moreover, inadequate supportive care may be an issue in low-resource settings which can contribute to the morbidity and mortality in some patients with DSS [2]. The high number of fatalities in DSS and its severity led us to study patient characteristics that were associated with mortality and prolonged hospital stay [3].

Several studies mentioned predictors for DSS mortality, including disseminated intravascular coagulation (DIC), massive bleeding, acute kidney injury (AKI), and acute liver injury (ALI) [5,8,13,14]. A study in Vietnam found that gender and elder age were associated with poorer outcomes [3]. A study in Pakistan in 2011 revealed that fluid overload percentage (FO%) was the second leading cause of death after the shock condition itself [7]. Studies on clinical outcomes of children with DSS treated in the PICU are still limited while findings from studies in other settings may not be applicable in our setting. The wide practice of unsafe traditional medications in combination with limited access to PICU facilities may have implications for the care of children with DSS in the Indonesian setting. Furthermore, the complexity of the referral system for critically ill children are often cause delays in treatment. This study aimed to evaluate the clinical outcomes of patients with DSS admitted to our PICU and its associated factors.

## 2 Methods

### 2.1 Study design and population

We retrospectively collected patients' data from January 2016 to May 2020 who had been diagnosed with DSS and admitted to the PICU in our tertiary hospital (Dr. Sardjito Hospital, Yogyakarta, Indonesia). As a tertiary referral hospital, we receive patients who are referred from primary and secondary hospitals (previous hospital) [25]. Establishment of DSS diagnosis of patients involved in this study was reevaluated for the serological and or NS-1 presence, and any presence of hemodynamic instability of the following: (1) rapid pulse and weak with narrow pulse pressure <20 mmHg, (2) systolic blood pressure less than 5th percentile according to the age with hypoperfusion clinical manifestation such as decrease of urine output (diuresis less than 1 ml/kg/hour in a patient whose body weight <30 kg or diuresis <0.5 ml/kg/hour in a patient whose body weight > 30 kg), (3) decrease of consciousness, and (4) cold or clammy skin [15]. Included patients were those who were admitted to PICU with DSS. All patients were followed-up until they reached death as an outcome or date of PICU discharge. Patients with missing medical records or incomplete data related with DSS criteria and evaluated factors were excluded from the analysis.

### 2.2 Data collection

We recorded all demographic, clinical and laboratory data of all patients during their admission in PICU. The demographic data that we evaluated were age and gender. Clinical data were: nutritional status, day of fever, hematology profile, PICU length of stay (LOS), shock event, shock duration, history of fluid resuscitation, inotropic use, blood transfusion, ventilator use, CVC use, FO%, prolonged shock, DIC, AKI.

Patients’ nutritional status was classified according to the World Health Organization (WHO) growth charts: Weight-for-Height curve for children younger than 5 years old or body mass index (BMI)-for-age curve for children 5 years old or older. Children were categorized into having good nutritional status (−2 ≤ z < 2 SD), being under-nourished (z < −2 SD) or overweight (z > 2 SD) [20]. We identified the subjects' age based on birth date on their ID card [22]. A live birth within the first year (<365 days) is defined as an infant age [27]. Shock condition at PICU admission was defined by shock condition when patient reached PICU admission, and patients were classified as “shock” and “no shock” [23]. DIC is a systemic syndrome with enhanced intravascular fibrin formation and deposition causing coagulation [[18], [21]]. We used the International Society on Thrombosis and Hemostasis (ISTH) diagnostic criteria to diagnose DIC patients [24]. AKI was determined by Pediatric Risk, Injury, Failure, Loss, End-Stage Renal Disease criteria [20].

FO% was calculated based on the total fluid input minus total fluid output divided by body weight at hospital admission (in kilograms) and multiplied by 100%. FO% was calculated since the first 24 h after PICU admission until they reached PICU outcome [14]. Patients were classified as having FO% > 10%” and “FO% ≤10%”.

### 2.3 Outcome measurement

For every patient, the follow-up period ended when the outcome, death or PICU discharge was reached. The mortality rate of DSS was defined as the proportion of patients who died during PICU stay among all children with DSS who were admitted to PICU within the study period. The secondary outcome was prolonged stay, which defined as length of stay (LOS) in the PICU ≥7 days starting from PICU admission until discharge, transfer to the ward, or deceased [26].

### 2.4 Data analysis

Categorical variables are presented as counts and percentages. Continuous data are presented as mean and standard deviation (SD) for normally distributed data or median and quartiles (Q) Q1 and Q3 for skewed data. Logistic regression was performed in pediatric patients admitted to the PICU, with observation time starting at the time of diagnosis of DSS (whether at admission or later during admission) and ending at the time of outcome occurrence (decease or survive) or the study closing date, to evaluate which characteristics could be associated with patients’ outcome. If p values in univariable analysis were <0.05, then the variables were included into a multivariable analysis to estimate the mutually independent associations between selected characteristics and outcomes. Data were analyzed using IBM SPSS Statistics 23rd version (IBM Corp., Chicago).

### 2.5 Ethics approval

This study was approved by the Medical and Health Research Ethics Committee of the Faculty of Medicine, Public Health and Nursing, Universitas Gadjah Mada, Yogyakarta, Indonesia (KE/FK/0024/EC/2020). The study was registered at the “Research Registry” with unique identification number UIN of 6861. This study has been reported in line with the Strengthening the Reporting of Cohort Studies in Surgery (STROCSS) criteria [27].

## 3 Results

### 3.1 Patient characteristics

Characteristics of patients with DSS are shown in Table 1. Overall, there were 55.6% males and 44.4% females. Most subjects were childhood age (56.15%). Of 146 patients, more than half (72.6%) had good nutritional status, while overweight and undernourished were 17.8% and 9.6%, respectively. The median day of fever when admitted to our PICU was day 5th of fever (day 3rd-8th). Most patients had FO ≤ 10% (56.8%). A low thrombocyte profile was commonly found with a median of 19 (12–30) (x10^3^/μL) at the first laboratory examination in our hospital.

**Table 1:** Characteristics of pediatric patients with dengue shock syndrome.

	Deceased (n = 8)	Survived (n = 138)	Total (n = 146)
Age group, n (%)			
Infant	3 (37.5%)	13 (9.4%)	16 (11%)
1–5 years old	2 (25%)	46 (33.3%)	48 (32.9%)
>5 years old	3 (37.5%)	79 (57.2%)	82 (56.1%)
Gender, n (%)			
Male	2 (25%)	76 (55.1%)	78 (53.4%)
Nutritional status			
Under nourished	1 (12.5%)	13 (9.4%)	14 (9.6%)
Normal	5 (62.5%)	101 (73.1%)	106 (72.6%)
Overweight	2 (25%)	24 (17.3%)	26 (17.8%)
Day of fever, median ± IQR	6 ± 5	5 ± 5	5 ± 5
Hematology profile, median ± IQR			
Highest hematocrite (%)	44.5 ± 15.52	46.8 ± 7.50	46.1 ± 7.50
Lowest thrombocyte (x10^3^/μL)	12 ± 8.3	20 ± 20	19 ± 18.5
Lowest leucocyte (x10^3^/μL)	4.58 ± 5.53	4.16 ± 2.36	4.16 ± 2.54
Length of stay (hour) in previous hospital, median ± IQR	28.5 ± 76.25	8 ± 23	8 ± 24
Shock event at emergency unit, n(%)	2 (25%)	45 (32.6%)	47 (32.2%)
History of fluid resuscitation			
>40 ml/kg in 1 period, n (%)	3 (37.5%)	26 (18.8%)	29 (19.8%)
Inotropic use ≥ 2, n (%)	1 (12.5%)	119 (86.2%	120 (82.2%)
Blood transfusion, n (%)			
PRC	6 (75%)	14 (10.1%)	20 (13.7%)
TC	7 (87.5%)	41 (29.7%)	48 (32.9%)
FFP	3 (37.5%)	22 (15.9%)	25 (17.1%)
Ventilator use, n (%)			
Yes	8 (100%)	24 (17.4%)	32 (21.9%)
No	0 (0%)	114 (82.6%)	114 (78.1%)
CVC use, n (%)			
Yes	4 (50%)	18 (13.0%)	22 (15.1%)
No	4 (50%)	120 (86.9%)	124 (84.9%)

AKI: acute kidney injury; CVC: central vein catheter; DIC: disseminated intravascular coagulation; FFP: fresh frozen plasma; IQR: interquartile range; PRC: packed red cell; TC: thrombocyte concentrate; WB: whole blood.

Almost one-third of patients (32.2%) diagnosed of having shock condition during admission in our emergency unit. While LOS at the previous hospital was not different between the two groups.

During treatment, the majority of patients did not need more than two vasoactive drugs. Most patients received a blood transfusion with thrombocyte concentrate being the most common blood component that patients received. About one-fifth of patients with DSS who were admitted to PICU needed mechanical ventilation support and a central venous catheter (CVC).

### 3.2 Clinical outcomes

A chi-squared test could not be applied since there was a cell count of less than five. Bivariate analysis showed that FO%, shock condition at PICU admission, DIC, AKI and infant age had a significant correlation with DSS mortality. Nutritional status was not related to DSS mortality based on that bivariate analysis.

Cross-tabulation between predictor variables and the outcome variable had zero cases in one cell, thus in relative risk (RR) statistical calculation, it should be added with 0.5 on each cell. Bivariate analysis revealed that FO% had 11.7 times higher risk for DSS mortality (95% CI: 1.53–90.57). Other variables with significant correlation were prolonged shock, DIC, AKI, and infant age, as presented in Table 2.

**Table 2:** Bivariate analysis for DSS mortality.

Predictor variable	Deceased (n = 8)	Survived (n = 138)	*p*	RR	95% CI
Fluid status					
FO% >10%	8 (12.7%)	55 (87.3%	<0.01	11.77	1.53–90.57
No FO% >10%	0 (0.0%)	83 (100.0%)			
Shock at PICU admission					
Yes	5 (71.4%)	2 (28.6%)	<0.01	33.09	9.84–111.36
No	3 (2.2%)	136 (97.8%)			
DIC					
Yes	7 (29.2%)	17 (70.8%)	<0.01	35.58	4.59–276.15
No	1 (0.8%)	121 (99.2%)			
Age					
Infant	3 (18.8%)	13 (81.3%)	0.043	4.88	1.28–18.50
Beyond infant age	5 (3.8%)	125 (96.2%)			
Nutritional status					
Overweight and obese	2 (7.7%)	24 (92.3%)	0.633	1.54	0.33–7.19
Not overweight/obese	6 (5.0%)	114 (95%)			

CI: Confidence interval; DIC: Disseminated intravascular coagulation; PICU: Pediatric Intensive Care Unit; RR: Relative risk.

Predictor variables with *p*-value < 0.05 were further analyzed with multivariate logistic regression analysis to determine the independent variables that can predict DSS mortality. However, due to the presence of zero cases, the multivariate analysis could not be applied. Instead, the backward method could still be used to determine the predictor variables that are significantly related to DSS mortality. Age group was excluded and this revealed the predictor variables as seen in Table 3.

**Table 3:** Backward method on logistic regression test.

Variable	Significance of the Change	
	Step 1	Step 2
Fluid overload	.009	.011
Prolonged shock	.000	.001
DIC	.000	.000
AKI	.006	.007
Infant age	.306	Excluded

AKI: Acute kidney injury; DIC: Disseminated intravascular coagulation.

Four predictors had *p-*value <0.05 towards the length of PICU stay (Table 4), which were fluid overload percentage, DIC, and AKI. Further multivariate analysis revealed that fluid overload was independent predictors of prolonged PICU stay.

**Table 4:** Bivariate and multivariate analysis for length of PICU stay outcome.

Predictor variable	Length of PICU stay		Bivariate analysis			Multivariate analysis		
	Prolonged PICU stay (n = 32)	No prolonged PICU stay (n = 114)	*p*	RR	95% CI	*p*	RR	95% CI
Fluid status at 48 h PICU								
Fluid overload	9 (14.3%)	54 (85.7%)	0.03	3.95	1.12–14.0	0.29	0.34	0.12–0.97
No fluid overload	3 (3.6%)	80 (96.4%)						
Shock at PICU admission								
Shock	1 (14.3%)	6 (85.7%)	0.46	1.81	0.27–12.08			
No shock	11 (7.9%)	128 (92.1%)						
DIC								
Yes	8 (33.3%)	16 (66.7%)	<0.01	10.17	3.33–31.08	<0.01	15.26	4.97–46.81
No	4 (3.3%)	118 (96.7%)						
AKI								
Yes	3 (21.4%)	11 (78.6%)	0.09	3.14	0.96–10.28	0.63	0.65	0.44–0.95
No	9 (6.8%)	123 (93.2%)						
Age								
Infant	2 (12.5%)	14 (87.5%)	0.62	1.63	0.39–6.77			
Beyond infant age	10 (7.7%)	120 (92.3%)						
Nutritional status								
Overweight and obese	7 (26.9%)	19 (73.1%)	<0.01	6.46	2.22–18.77	<0.01	16.47	3.72–72.9
No overweight/obese	5 (4.2%)	115 (95.8%)						

AKI: Acute kidney injury; CI: Confidence interval; DIC: Disseminated intravascular coagulation; PICU: Pediatric Intensive Care Unit; RR: Relative risk.

## 4 Discussion

We found that FO% > 10%, shock condition at PICU admission, DIC and AKI in children with DSS were associated with mortality. Moreover, DIC and nutritional state were associated with prolonged PICU stay.

The study was located in one of the referral hospitals equipped with a tertiary-care PICU facility, that particularly patients with DSS are referred to. There were some limitations to the consideration of our study. Our setting still uses manual medical records. We tried to minimize all data collection error by adding a second data collector who validated the data input process. This study was also a single-center study, that might not reflect the overall situation of pediatric patients with DSS in Indonesia.

FO% > 10% was the most common complication found in patients with DSS treated in our setting. Comparing with the previous cohort retrospective study in 2011–2017, there was an increasing percentage of patients with DSS whose FO% from 16% to 37.7% [14]. More than one-third of the DSS cases had signs of FO% > 10% at the time they arrived at Dr. Sardjito hospital. Measurement of fluid status at 48 h PICU stay revealed that 52.4% among subjects with FO% > 10% had previously experienced signs of FO% > 10% before PICU admission.

A study in 2019 with prospective design in the PICU at Dr. Sardjito General Hospital Indonesia showed that most of the patients with DSS are referrals with a complex case and comorbidities. Evaluations with ultrasound cardiac output monitoring (USCOM) resulted in a low inotropic index and high systemic vascular resistance [[9], [11]]. Even though risk factors for fluid overload on baseline characteristics were not different between groups, however, most of these patients with DSS suffered from hemodynamic disturbance. Organ dysfunction was found more in the FO% > 10% group.

These study findings were similar to a retrospective cohort study in Sri Lanka in 2018 which stated that fluid restriction, including resuscitation or maintenance, was not sufficient to prevent FO% > 10%. In severe dengue, permeability disruption has a role in fluid accumulation into tissues and organs [[16], [17]]. Organ dysfunction due to fluid accumulation underlies medical invasive use such as a ventilator [18].

More than half of the patients with DSS were beyond 5 years old, which were 82 of 146 cases (56.1%), with a median age of 6 years old. According to a 45 years’ dengue report which was reported in 2018, the majority of dengue infections were found in the 5–14 years old age group, and DSS as one clinical spectrum was found more in this age group [18]. Infant age was found significantly more in the deceased group. Several complex hypotheses regarding these findings were contributing to each other, which include more permeable vascular physiology, lower immune system, and presence of maternal memory cell rand T cell regulator. Transmitted T cell memory induces cytokine storm when the baby is primarily infected by dengue, while transmitted T cell regulator causes immunosuppression that leads to dengue viral replication. That role of maternal T cell memory is the main cause of severe plasma leakage and thus, FO% > 10% was commonly found in this study [4].

Bivariate and multivariate analyses revealed a significant correlation between FO > 10% towards DSS mortality, and also the prolonged shock, DIC, and AKI variables. It was previously concluded that the presence of difficulties or comorbidities can predict mortality in patients with DSS [8,10,13]. Patients who survived from DIC require more medical interventions, including blood transfusions, vasopressors and mechanical ventilation. This causes DIC sufferers to require longer treatment and have greater probability to decease [6,12]. Infant age group was automatically excluded from multivariate analysis with a backward method. This revealed that mortality in this age group occurred by another variable besides infant age, as did the nutritional status of the patient that did not become an independent variable for DSS mortality.

Both the fluid resuscitation and blood transfusion in DIC condition influenced the presence of FO% > 10%, as well as AKI condition. A retrospective study from January 2007 to December 2013 in critically ill patients with AKI showed that improper fluid management has an HR 1.03 (95% CI: 1.4–1.13). In this population, FO>10% was found more in sepsis condition (*p* < 0.001), however, it was not statistically proven with HR 1.16 (95% CI: 0.86–1.56) [6].

FO% > 10% was a risk factor of prolonged ventilator use and so does interfere with PICU stay. Fluid accumulation on lung parenchyma causes increasing in PIP and PEEP [18]. That finding, however, was a result of a study with critically ill patients, not for the DSS population. A retrospective cohort study that involved 154 children in the DSS population in 2019 showed no difference in length of ventilator use and length of PICU stay among those who received liberal or restrictive fluid therapy, however, that study did not reveal fluid status of every subject and whether they had FO% > 10% or not [19].

Infection and inflammatory mediators lead to more severe glycocalyx that leads to organ dysfunction and prolonged PICU stay. FO% > 10% is a preventable complication by proper fluid management and strict monitoring of fluid balance. Early detection of fluid overload is expected to decrease morbidity and mortality in patients with DSS and reduce the length of PICU stay.

## 5 Conclusion

FO% > 10%, DIC, AKI and shock condition at PICU admission associated to DSS mortality. DIC and nutritional status associated to prolonged PICU stay.

## Funding

This research did not receive any specific grant from funding agencies in the public, commercial, or not-for-profit sectors.

## Ethical approval

This study has been approved by the Ethical Committee of Faculty of Medicine, Public Health and Nursing, Universitas Gadjah Mada/Dr. Sardjito Hospital (Ref: KE/FK/0024/EC/2020).

## Please state any sources of funding for your research

The authors declare that this study had no funding source.

## Author contribution

Syifa Armenda, Desy Rusmawatingintyas, and Eggi Arguni conceived the study and critically revised the manuscript for important intellectual content. Firdian Makrufardi drafted the manuscript and critically revised the manuscript for important intellectual content. All authors read and approved the final draft. All authors facilitated all project-related tasks.

## Registration of research studies

researchregistry6861

https://www.researchregistry.com/browse-the-registry#home/registrationdetails/60af19a4235ed6001fbd98b1/

## Guarantor

Desy Rusmawatiningtyas

## Consent

Written informed consent was obtained from the patient for publication of this study and accompanying images. A copy of the written consent is available for review by the Editor-in-Chief of this journal on request.

## Provenance and peer review

Not commissioned, externally peer reviewed.

## Declaration of competing interest

No potential conflict of interest relevant to this article was reported.
